# Associations of pre-hospital statin treatment with in-hospital outcomes and severity of coronary artery disease in patients with first acute coronary syndrome-findings from the CCC-ACS project

**DOI:** 10.3389/fcvm.2022.1030108

**Published:** 2023-01-18

**Authors:** Lisha Zhang, Yan Zhang, Chao Chu, Fuxue Deng, Juan Zhou, Zuyi Yuan

**Affiliations:** ^1^Department of Cardiovascular Medicine, The First Affiliated Hospital of Xi’an Jiaotong University, Xi’an, Shaanxi, China; ^2^Department of Cardiovascular Medicine, The Second Affiliated Hospital of Xi’an Jiaotong University, Xi’an, Shaanxi, China

**Keywords:** acute coronary syndrome, in-hospital major adverse cardiovascular event, pre-hospital statin treatment, low-density lipoprotein cholesterol, coronary artery disease prognostic index

## Abstract

**Background:**

The current burden of dyslipidemia, the pre-hospital application of statins and the association of pre-hospital statins with the severity of coronary artery disease (CAD) and in-hospital outcomes in Chinese patients with first acute coronary syndrome (ACS) are very significant and remain unclear.

**Methods:**

A total of 41,183 patients who underwent coronary angiography and were diagnosed with ACS for the first time from a nationwide registry study (CCC-ACS) were enrolled. The severity of CAD was assessed using the CAD prognostic index (CADPI). The patients were classified into statin and non-statin groups according to their pre-hospital statin treatment status. Clinical characteristics, CADPI and in-hospital outcomes were compared, and a logistic regression analysis was performed to determine whether pre-hospital statin therapy is associated with in-hospital outcomes and CADPI. A sensitivity analysis was used to further explore the issues above.

**Results:**

The non-statin group had more in-hospital all-cause deaths (1.2 vs. 0.8%, *P* = 0.010). However, no association exists between statin pretreatment and in-hospital major adverse cardiovascular events (MACEs) or all-cause deaths in the entire population and subgroups (all *P* > 0.05). Surprisingly, statin pretreatment was associated with an 8.9% higher risk of severely obstructive CAD (CADPI ≥ 37) (OR, 1.089; 95% CI, 1.010–1.175, *P* = 0.028), and similar results were observed in subgroups of females, those aged 50 to 75 years, and patients with hypertension.

**Conclusion:**

Statin pretreatment was not related to MACEs or all-cause death during hospital stay, but it was associated with a higher risk of increased angiographic severity in patients with first ACS.

## 1. Introduction

Coronary artery disease (CAD) remains the leading cause of mortality worldwide (1, 2). Moran et al. (3) reported that China is predicted to experience a 69% increase in the incidence of CAD in 2020–2029 compared with 2000–2009. Acute coronary syndrome (ACS) is a more severe and acute manifestation of CAD.

Dyslipidemia is an independent risk factor for CAD, and statin therapy is well established for the primary and secondary prevention of cardiovascular diseases. Each 1 mmol/L reduction in low-density lipoprotein cholesterol (LDL-C) with statin therapy reduces the risk of major adverse cardiovascular events (MACEs) by approximately one-fifth (4–7). Hence, the current burden of dyslipidemia in patients with ACS and the pre-hospital application of statins among Chinese patients with ACS should be evaluated.

Some studies have investigated the association of statin pretreatment with cardiovascular outcomes, but the results remain controversial. Ndrepepa et al. found that pretreatment of statins before percutaneous coronary intervention (PCI) was associated with a significant reduction of 1-year mortality in patients with stable angina (8). Two meta-analysis of fifteen randomized controlled trials found that high-dose statin pretreatment can significantly reduce MACE after PCI (9–11). However, another study from Anzhen Hospital found that preoperative statin therapy did not reduce the risk of mortality and MACCE during the hospital stay following successful PCI (12). And SECURE-PCI Randomized Clinical Trial also showed that among patients with ACS and planned invasive management with PCI, periprocedural loading doses of atorvastatin did not reduce the rate of MACE at 30 days (13). Therefore, the role of statin pretreatment therapy remains controversial.

In the current study, we investigated the pre-hospital application of statins and the current burden of dyslipidemia and determined the relationship between pre-hospital statin treatment and angiographic severity [evaluated with the CAD prognostic index (CADPI)] and in-hospital outcomes in patients with first ACS in a nationwide registry study with ‘real-world’ settings: the Improving CCC (Care for Cardiovascular Disease in China) ACS project (14).

## 2. Materials and methods

### 2.1. Study design and participants

CCC-ACS(NCT02306616), a national hospital-based quality improvement program, aims to increase adherence to ACS guidelines in China and to improve patient outcomes. Launched in 2014, the project had recruited 150 centers representing the diversity of care for ACS patients in tertiary hospitals across China at the time of the current study. For additional details, please refer to the published paper (14).

The inclusion criterion of the present study was the first occurrence of ACS, including unstable angina (UA), non-ST-segment elevation myocardial infarction (NSTEMI) and STEMI. The exclusion criteria were as follows: without CAG (coronary angiography) check, renal failure, past AMI, past PCI or CABG, history of stroke and heart failure, and lack of clarity about the application of pre-hospital statins, myocardial injury markers, severe clinical conditions at admission and in-hospital outcomes. After exclusion, 41,183 of 63,641 inpatients with first ACS were registered between April 2014 and June 2017 from 150 hospitals. This study was approved by the ethics committee of Beijing Anzhen Hospital, Capital Medical University.

### 2.2. Coronary artery disease prognostic index (CADPI)

The CADPI classification is hierarchical (0–100), and patients are assigned to the most severe category that applies to them. The CADPI cut-off point (≥37 versus <37) was selected to reflect > 70% stenosis in > 1 major epicardial coronary artery, which is a clinically actionable threshold for revascularization; this is also the cut-off point at which a survival benefit has been demonstrated previously for revascularization (15–17). Details are shown in Supplementary material (Supplementary Table 1).

### 2.3. In-hospital outcomes

The outcomes of this study included MACEs and all-cause deaths that occurred during hospitalization. MACEs were defined as a combination of cardiogenic death, recurrent myocardial infarction, stent thrombosis, and ischemic stroke.

### 2.4. Related definitions

STEMI and NSTEMI are defined for study inclusion according to the recommended diagnostic criteria of the STEMI guidelines of 2010 and the non–ST-elevation ACS (NSTE-ACS) guidelines of 2012 issued by the CSC (18, 19). Because the diagnosis criteria for UAP vary between hospitals, we defined the inclusion criteria for UAP in CCC-ACS as described in the published paper (14). Other clinical history of diseases, including cerebrovascular disease, heart failure, atrial fibrillation cardiac shock and cardiac arrest, was defined according to the notes on the original medical records. Hypertension was defined as having a history of hypertension or systolic blood pressure (SBP) ≥ 140 mmHg or diastolic blood pressure (DBP) ≥ 90 mmHg at admission. Elevated myocardial injury markers (five-fold) (including troponin I, troponin T or creatine kinase isoenzymes-MB (CK-MB)) were defined as levels higher than five-fold of the upper reference limit (20). Current smoking was defined as smoking in the preceding 1 year according to the medical records of the patients. Dyslipidemia: total cholesterol level of 6.2 mmol/L (240 mg/dL) or higher, or low-density lipoprotein cholesterol level of 4.1 mmol/L (160 mg/dL) or higher when presenting at the hospital. The lipid-lowering treatment goals recommended by AHA/ACC in 2018 were LDL-C target levels < 1.8 mmol/L (70 mg/dl) for patients with arteriosclerotic cardiovascular disease (ASCVD) (21). Pre-hospital medicine treatment was defined as receiving statins pre-admission (two weeks before admission), including all types of statins. All these data, including a history of diabetes and other medical history, were obtained from the patients’ medical records.

### 2.5. Statistical analysis

Statistical analyses were performed using SPSS 23.0 (SPSS Inc., Chicago, IL). Continuous variables were summarized using the mean ± standard deviation (SD) or median (IQR), and categorical variables were presented by counts and percentages. Continuous variables between two groups were compared by the two-sided t-test or the Mann–Whitney U test according to the normality of the distribution. Continuous variables between three or more groups were compared by an ANOVA or the Kruskal-Wallis test according to the normality of the distribution. Categorical variables were assessed with the Chi-square test. We used Cox regression analysis to calculate HRs and 95% confidence intervals (CIs) for in-hospital outcomes comparisons. Cox regression were constructed based on a 15-day observation. Hazard ratios (HRs) for different variables and corresponding 95% confidence intervals (CIs) were reported. A binary logistic regression was performed to examine the association between statin pretreatment and in-hospital outcomes or CADPI. Odds ratios (ORs) with 95% confidence intervals (CIs) were reported. Subgroup analyses of in-hospital outcomes and CADPI were then performed based on important characteristics, including age (younger than 50 years, between 50 and 75, or 75 years and older), sex (male or female), hypertension (no or yes), diabetes mellitus (DM, no or yes), PCI (no or yes), and Killip class (I-II or III-IV). Two-tailed p values of less than 0.05 were considered statistically significant.

## 3. Results

### 3.1. Baseline characteristics of patients with first ACS

A total of 5,224 (12.7%) patients with first ACS had a history of pre-hospital statin treatment. As showed in Table 1. The mean ages of the non-statin and statin groups were 59.7 ± 11.9 and 59.7 ± 11.3 years, respectively (*P* = 0.876). The statin group contained a higher proportion of females than the non-statin group (23.4 vs. 21.7%, *P* = 0.006). Fewer patients in the statin group had five-fold elevated myocardial injury markers than in the non-statin group (51.6 vs. 72.7%, *P* < 0.001).

**TABLE 1:** Clinical characteristics of patients.

Variables	Non-statin group *N* = 35,959	Statin group *N* = 5,224	*P*-value
Age (year)	59.7 ± 11.9	59.7 ± 11.3	0.876
**Age**			**0.008**
<50 (*n*, %)	7,202, 20.1	988, 19.0	
50 ≤ age < 75 (*n*, %)	24,540, 68.5	3,668, 70.6	
≥75 (*n*, %)	4,101, 11.4	543, 10.4	
Female (*n*, %)	7,811, 21.7	1,224, 23.4	0.006
Married (*n*, %)	33,540, 93.3	4,954, 94.8	<0.001
SBP (mmHg)	129.6 ± 22.8	129.0 ± 21.2	0.068
DBP (mmHg)	78.8 ± 14.2	77.6 ± 13.1	<0.001
Heart rate (bpm)	77.1 ± 15.3	75.1 ± 14.0	<0.001
Early family history of CAD (*n*, %)	177, 0.5	56, 1.1	<0.001
**Medical history**			
Current smoking (*n*, %)	17,472, 51.4	2,374, 45.4	<0.001
Hypertension (*n*, %)	16,756, 46.6	2,558, 49.0	0.001
Diabetes (*n*, %)	6,538, 18.2	1,099, 21.0	<0.001
Dyslipidemia (*n*, %)	2,095, 5.8	795, 15.2	<0.001
Atrial fibrillation (*n*, %)	416, 1.2	75, 1.4	0.088
**Laboratory test**			
Triglyceride (mmol/L)^a^	1.45 (1.03–2.12)	1.46 (1.08–2.08)	0.065
Total cholesterol (mmol/L)^b^	4.48 (3.79–5.25)	4.09 (3.38–4.83)	<0.001
LDL-C (mmol/L)^c^	2.76 (2.20–3.38)	2.43 (1.87–3.07)	<0.001
HDL-C (mmol/L)^d^	1.03 (0.87–1.24)	0.99 (0.84–1.19)	<0.001
Elevated LDL-C^c^ (*n*, %)	29,202, 89.9	3,779, 80.6	<0.001
Fast glucose (mmol/L)^e^	5.90 (5.05–7.62)	5.79 (4.93–7.48)	<0.001
Creatinine (μmoI/L)^f^	76.9 (65.0–90.1)	74.4 (63.6–87.6)	<0.001
Fivefold elevated myocardial injury marker (*n*, %)	26,130, 72.7	2,697, 51.6	<0.001
Killip class^g^			0.001
I(*n*, %)	25,278, 75.2	3,563, 74.0	
II(*n*, %)	6,381, 19.0	935, 19.4	
III(*n*, %)	1,098, 3.3	207, 4.3	
IV(*n*, %)	868, 2.6	110, 2.3	
**ACS type**			**<0.001**
STEMI (*n*, %)	24,738, 68.8	2,912, 55.7	
NSTEMI (*n*, %)	7,824, 21.8	1,184, 22.7	
UAP (*n*, %)	11,181, 31.1	1,128, 21.6	
**Severe clinical conditions at admission**			
HF (*n*, %)	1,997, 5.6	314, 6.0	0.187
Cardiac shock (*n*, %)	792, 2.2	108, 2.1	0.544
Cardiac arrest (*n*, %)	610, 1.7	82, 1.6	0.527
In-hospital PCI (*n*, %)	11,181, 31.1	2,340, 44.8	<0.001
**Pre-hospital medicine^h^**			
ACEI (*n*, %)	785, 2.2	781, 15.0	<0.001
ARB (*n*, %)	914, 2.5	468, 9.0	<0.001
β-blockers (*n*, %)	1,041, 2.9	1,571, 30.1	<0.001
**In-hospital medicine ^i^**			
DAPT (*n*, %)	33,813, 94.0	4,930, 94.9	0.246
ACEI (*n*, %)	3,891, 10.8	668, 12.8	<0.001
ARB (*n*, %)	13,654, 38.0	1,881, 36.0	0.007
β-blockers (*n*, %)	20,020, 55.7	3,039, 58.2	<0.001
Statin (*n*, %)	33,683, 93.8	4,932, 94.6	0.026
Hospital stays (day)	9 (7–12)	9 (7–12)	<0.001

Values are mean ± SD, median (IQR), or *n* (*n*, %). SBP, systolic blood pressure; DBP, diastolic blood pressure; CAD, coronary artery disease; HF, heart failure; LDL-C, low-density lipoprotein cholesterol; HDL-C, high-density lipoprotein cholesterol; ACS, acute coronary syndromes; STEMI, ST-segment elevation myocardial infarction; NSTEMI, non-ST-segment elevation myocardial infarction; UAP, unstable angina pectoris; PCI, percutaneous coronary intervention; ACEI, angiotensin-converting enzyme inhibition; ARB, angiotensin receptor blocker; DAPT, dual antiplatelet therapy; Elevated LDL-C: LDL-C ≥ 1.8 mmol/L. A value of *P* < 0.05 was considered statistically significant.

^a^Data of triglyceride were not available for 3,670 patients.

^b^Data of total cholesterol were not available for 3,803 patients.

^c^Data of LDL-C were not available for 3,994 patients.

^d^Data of HDL-C were not available for 3,845 patients.

^e^Data of fast glucose were not available for 3,808 patients.

^f^Data of creatinine not available for 1,296 patients.

^g^Data of Killip class were not available for 2,743 patients.

^h^Data of pre-hospital medicine were not available for 8 patients.

^i^Data of in-hospital medicine were not available for 49 patients.

### 3.2. Lipid status in patients with first ACS

As shown in Table 1, the LDL-C levels were significantly lower in the statin group than in the non-statin group [2.43 (1.87–3.07) vs. 2.76 (2.20–3.38) mmol/L, *P* < 0.001]. A total of 29,202 (89.9%) and 3,779 (80.6%) patients in the non-statin and statin groups (*P* < 0.001) did not meet the lipid-lowering treatment goals [LDL-C < 1.8 mmol/L (70 mg/dl)] for patients with arteriosclerotic cardiovascular disease (ASCVD), respectively. Similar to LDL-C, total cholesterol levels were also further decreased in the statin group compared with the non-statin group [4.09 (3.38–4.83) vs. 4.48 (3.79–5.25) mmol/L, *P* < 0.001]. However, pre-hospital statin treatment did not increase the HDL-C levels [statin vs. non-statin: 0.99 (0.84–1.19) vs. 1.03 (0.87–1.24) mmol/L, *P* < 0.001] and did not result in any differences in triglycerides between the 2 groups [statin vs. non-statin: 1.46 (1.08–2.08) vs. 1.45 (1.03–2.12) mmol/L, *P* = 0.065].

### 3.3. In-hospital outcomes in patients with ACS

**FIGURE 1:**
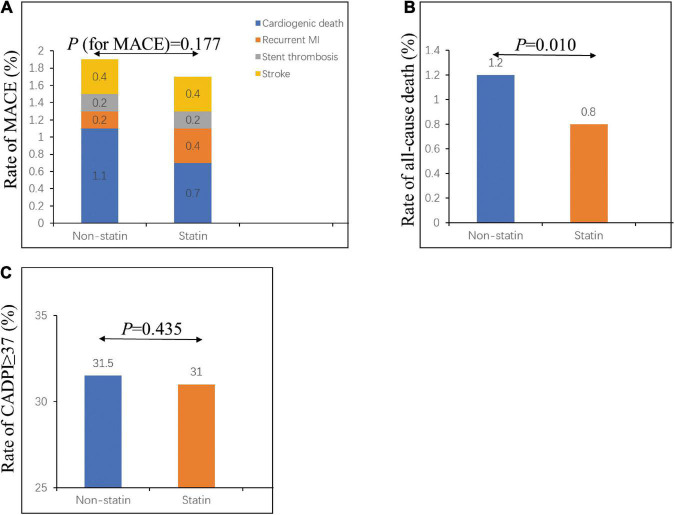
In-hospital outcomes and severely obstructive CAD (CADPI ≥ 37) in patients with first ACS. **(A)** Rate of in-hospital MACE in the non-statin and statin groups. **(B)** Rate of in-hospital all-cause death in the non-statin and statin groups. **(C)** Rate of CADPI ≥ 37 in the non-statin and statin groups. CADPI, coronary artery disease prognostic index; MACE, major adverse cardiovascular event. A value of *P* < 0.05 was considered statistically significant.

As shown in Table 2 and Figure 1, the percentage of patients with severely obstructive CAD (CADPI ≥ 37) did not differ between the two groups (*P* = 0.435). A total of 473 patients died during hospitalization (1.2% in the non-statin group and 0.8% in the statin group, *P* = 0.010). However, the rates of MACEs did not differ between the two groups (1.8 vs. 1.5%, *P* = 0.177).

**TABLE 2:** In-hospital clinical outcomes and CADPI of patients with first ACS.

Variables	Non-statin group *N* = 33,962	Statin group *N* = 4,910	*P*-value
CADPI score ≥ 37 (*n*, %)	11,338, 31.5	1,619, 31.0	0.435
All-cause death (*n*, %)	432, 1.2	41, 0.8	0.010
Overall MACE (*n*, %)	648, 1.8	80, 1.5	0.177
Cardiogenic death	410, 1.1	37, 0.7	0.005
Recurrent MI	78, 0.2	21, 0.4	0.015
Stent thrombosis	56, 0.2	8, 0.2	1.000
Stroke	150, 0.4	19, 0.4	0.644

Values are mean ± SD, median (IQR), or *n* (*n*, %). CADPI, coronary artery disease prognostic index; MACE, major adverse cardiovascular event; MI, myocardial infarction. A value of *P* < 0.05 was considered statistically significant.

### 3.4. Independent risk factors of in-hospital MACE and severely obstructive CAD (CADPI ≥ 37)

To further explore the effect of pre-hospital statin treatment on in-hospital outcomes in patients with first ACS, we conducted a logistic regression analysis. As shown in Table 3, pre-hospital statin treatment showed no association with in-hospital MACE (odds ratio (OR), 0.991; 95% confidence interval (CI), 0.722–1.360, *P* = 0.955) and all-cause death (OR, 0.970; 95% CI, 0.611–1.540, *P* = 0.898) after controlling for confounders. Surprisingly, statin pretreatment was associated with an 8.9% higher risk of severely obstructive CAD (CADPI ≥ 37) (OR, 1.089; 95% CI, 1.010–1.175, *P* = 0.028). In addition, a Cox regression about MACE/all-cause death was employed based on the in-hospital stay (within 15 days) and statin pretreatment and the results showed no association with MACE/all-cause death (HR, 0.941; 95% CI, 0.662–1.340, *P* = 0.738 and HR, 0.914; 95% CI, 0.564–1.483, *P* = 0.716, respectively) after controlling for confounders (Table 4).

**TABLE 3:** Independent predictors of in-hospital MACE, all-cause death and severely obstructive CAD (CADPI ≥ 37).

Variables	Adjusted OR (95% CI)^(a)^	*P*-value^(a)^	Adjusted OR (95% CI)^(b)^	*P*-value^(b)^
**(1) For MACE**				
Age	1.050 (1.042–1.057)	<0.001	1.037 (1.028–1.046)	<0.001
Male	0.766 (0.652–0.900)	0.001	0.824 (0.658–1.032)	0.092
Current smoking			0.846 (0.685–1.046)	0.122
Early family history of CAD			0.995 (0.244–4.054)	0.994
Hypertension			0.916 (0.755–1.112)	0.377
DM			1.043 (0.830–1.311)	0.718
**ACS type**				
STEMI				0.028
NSTEMI			0.503 (0.290–0.875)	0.015
UAP			0.696 (0.556–0.872)	0.002
**Killip class**				
I				0.543
II			1.067 (0.842–1.353)	0.591
III			0.894 (0.583–1.371)	0.607
IV			1.282 (0.845–1.946)	0.242
HR			1.016 (1.011–1.021)	<0.001
**Severe clinical conditions at admission**				
Cardiac shock			1.888 (1.246–2.861)	0.003
Cardiac arrest			4.046 (2.814–5.817)	<0.001
HF			1.953 (1.481–2.577)	<0.001
**Pre-hospital medicine**				
β-blockers			1.334 (0.877–2.030)	0.178
ACEI			1.090 (0.641–1.855)	0.750
ARB			1.373 (0.817–2.307)	0.231
Statin	0.858 (0.679–1.085)	0.202	0.991 (0.722–1.360)	0.955
**Laboratory test**				
Fivefold elevated myocardial injury marker			1.698 (1.281–2.249)	<0.001
Creatinine (μmoI/L)			1.005 (1.003–1.006)	<0.001
LDL-C ≥ 1.8			1.279 (0.978–1.672)	0.072
**In-hospital medicine**				
β-blockers			0.883 (0.721–1.083)	0.232
ACEI			0.732 (0.512–1.046)	0.087
ARB			0.813 (0.654–1.010)	0.062
Statin			1.023 (0.692–1.513)	0.908
DAPT			0.723 (0.508–1.030)	0.073
In-hospital PCI			0.696 (0.556–0.872)	0.002
**(2) For all-cause death**				
Age	1.069 (1.060–1.079)	<0.001	1.056 (1.044–1.069)	<0.001
Male	0.674 (0.555–0.817)	<0.001	0.655 (0.489–0.876)	0.004
Current smoking			0.812 (0.601–1.099)	0.177
Early family history of CAD			1.372 (0.188–9.985)	0.755
Hypertension			0.808 (0.619–1.054)	0.116
DM			1.277 (0.948–1.721)	0.107
**ACS type**				
STEMI				0.026
NSTEMI			0.860 (0.622–1.190)	0.364
UAP			0.151 (0.036–0.630)	0.010
**Killip class**				
I				0.060
II			1.443 (1.055–1.974)	0.022
III			0.842 (0.478–1.485)	0.553
IV			1.319 (0.788–2.208)	0.291
HR			1.024 (1.017–1.030)	<0.001
**Severe clinical conditions at admission**				
Cardiac shock			2.065 (1.246–3.422)	0.005
Cardiac arrest			6.417 (4.199–9.806)	<0.001
HF			1.792 (1.266–2.535)	0.001
**Pre-hospital medicine**				
β-blockers			0.868 (0.417–1.807)	0.705
ACEI			0.887 (0.370–2.125)	0.787
ARB			0.822 (0.347–1.948)	0.656
Statin	0.665 (0.482–0.918)	0.013	0.970 (0.611–1.540)	0.898
**Laboratory test**				
Fivefold elevated myocardial injury marker			1.667 (1.121–2.478)	0.012
Creatinine (μmoI/L)			1.006 (1.004–1.007)	<0.001
LDL-C ≥ 1.8			1.253 (0.865–1.817)	0.233
**In-hospital medicine**				
β-blockers			0.746 (0.565–0.986)	0.040
ACEI			1.030 (0.650–1.633)	0.899
ARB			0.851 (0.630–1.149)	0.291
Statin			0.952 (0.557–1.625)	0.856
In-hospital PCI			0.367 (0.248–0.542)	<0.001
**(3) For CADPI**				
Age	1.000 (0.998–1.002)	0.990	1.001 (0.999–1.004)	0.168
Male	1.150 (1.090–1.212)	<0.001	1.102 (1.035–1.173)	0.003
Current smoking			1.040 (0.989–1.094)	0.127
Early family history of CAD			1.490 (1.119–1.985)	0.006
Hypertension			1.089 (1.039–1.142)	<0.001
DM			1.163 (1.097–1.233)	<0.001
**ACS type**				
STEMI				<0.001
NSTEMI			0.850 (0.804–0.900)	<0.001
UAP			0.496 (0.454–0.543)	<0.001
**Killip class**				
I				<0.001
II			1.133 (1.070–1.201)	<0.001
III			0.971 (0.854–1.104)	0.651
IV			1.146 (0.988–1.329)	0.072
HR			1.005 (1.003–1.006)	<0.001
**Pre-hospital medicine**				
β-blockers			1.066 (0.962–1.182)	0.221
ACEI			1.031 (0.911–1.167)	0.629
ARB			0.938 (0.820–1.072)	0.349
Statin	0.978 (0.918–1.042)	0.490	1.089 (1.010–1.175)	0.028
LDL-C ≥ 1.8			0.723 (0.670–0.780)	<0.001
In-hospital statin			1.029 (0.935–1.132)	0.558

CAD, coronary artery disease; DM, diabetes mellitus; ACS, acute coronary syndromes; STEMI, ST-segment elevation myocardial infarction; NSTEMI, non-ST-segment elevation myocardial infarction; UAP, unstable angina pectoris; HR, heart rate; HF, heart failure; ACEI, angiotensin-converting enzyme inhibition; ARB, angiotensin receptor blocker; LDL-C, low-density lipoprotein cholesterol; DAPT, dual antiplatelet therapy; CADPI, Coronary Artery Disease Prognostic Index; MACE, major adverse cardiovascular event. A value of *P* < 0.05 was considered statistically significant.

Model (a):

Adjusted variables included age, sex and pre-hospital statin.

Model (b):

Adjusted variables for MACE finally included age, sex, current smoking, early family history of CAD, hypertension, DM, ACS type, Killip class, HR, severe clinical conditions at admission (cardiac shock, cardiac arrest and HF), pre-hospital medicine (β-blockers, ACEI, ARB and statin), fivefold elevated myocardial injury marker, creatinine, LDL-C ≥ 1.8, in-hospital medicine (β-blockers, ACEI, ARB, statin and DAPT) and in-hospital PCI.

Adjusted variables for all-cause death finally included age, sex, current smoking, early family history of CAD, hypertension, DM, ACS type, Killip class, HR, severe clinical conditions at admission (cardiac shock, cardiac arrest and HF), pre-hospital medicine (β-blockers, ACEI, ARB and statin), fivefold elevated myocardial injury marker, creatinine, LDL-C ≥ 1.8, in-hospital medicine (β-blockers, ACEI, ARB and statin) and in-hospital PCI.

Adjusted variables for CADPI ≥ 37 finally included age, sex, current smoking, early family history of CAD, hypertension, DM, ACS type, Killip class, HR, pre-hospital medicine (β-blockers, ACEI, ARB and statin), LDL-C ≥ 1.8, and in-hospital statin.

**TABLE 4:** Cox regression analysis about in-hospital MACE or all-cause death in patients with first ACS based on the in-hospital stay (within 15 days).

	Variables	Adjusted HR (95% CI)	*P*-value
MACE	Pre-hospital statin	0.941 (0.662–1.340)	0.738
All-cause death	Pre-hospital statin	0.914 (0.564–1.483)	0.716

CAD, coronary artery disease; DM, diabetes mellitus; ACS, acute coronary syndromes; STEMI, ST-segment elevation myocardial infarction; NSTEMI, non-ST-segment elevation myocardial infarction; UAP, unstable angina pectoris; HR, heart rate; HF, heart failure; ACEI, angiotensin-converting enzyme inhibition; ARB, angiotensin receptor blocker; LDL-C, low-density lipoprotein cholesterol; DAPT, dual antiplatelet therapy; MACE, major adverse cardiovascular event. A value of *P* < 0.05 was considered statistically significant.

Adjusted variables for MACE finally included age, sex, current smoking, early family history of CAD, hypertension, DM, ACS type, Killip class, HR, severe clinical conditions at admission (cardiac shock, cardiac arrest and HF), pre-hospital medicine (β-blockers, ACEI, ARB and statin), fivefold elevated myocardial injury marker, creatinine, LDL-C ≥ 1.8, in-hospital medicine (β-blockers, ACEI, ARB, statin and DAPT) and in-hospital PCI.

Adjusted variables for all-cause death finally included age, sex, current smoking, early family history of CAD, hypertension, DM, ACS type, Killip class, HR, severe clinical conditions at admission (cardiac shock, cardiac arrest and HF), pre-hospital medicine (β-blockers, ACEI, ARB and statin), fivefold elevated myocardial injury marker, creatinine, LDL-C ≥ 1.8, in-hospital medicine (β-blockers, ACEI, ARB and statin) and in-hospital PCI.

**FIGURE 2:**
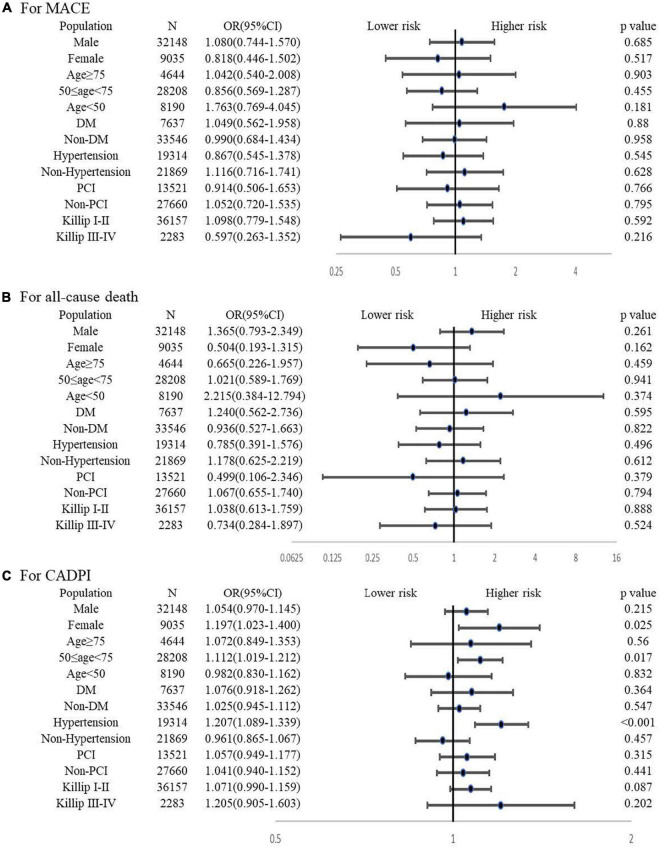
Subgroup analysis for the association between pre-hospital statin treatment and in-hospital outcomes or severely obstructive CAD (CADPI ≥ 37). ***(A)** Association between pre-hospital statin treatment and in-hospital MACE. **(B)** Association between pre-hospital statin treatment and in-hospital all-cause death. **(C)** Association between pre-hospital statin treatment and severely obstructive CAD (CADPI ≥ 37). DM, diabetes mellitus; PCI, percutaneous coronary intervention; CADPI*, coronary artery disease prognostic index*; MACE, major adverse cardiovascular event. A value of* P < *0.05 was considered statistically significant. Adjusted variables for MACE finally included age, sex, current smoking, early family history of CAD, hypertension, DM, ACS type, Killip class, HR, severe clinical conditions at admission (cardiac shock, cardiac arrest and HF), pre-hospital medicine (β -blockers, ACEI, ARB and statin), fivefold elevated myocardial injury marker, creatinine, LDL-C ≥ 1.8, in-hospital medicine (β -blockers, ACEI, ARB, statin and DAPT) and in-hospital PCI. Adjusted variables for all-cause death finally included age, sex, current smoking, early family history of CAD, hypertension, DM, ACS type, Killip class, HR, severe clinical conditions at admission (cardiac shock, cardiac arrest and HF), pre-hospital medicine (β -blockers, ACEI, ARB and statin), fivefold elevated myocardial injury marker, creatinine, LDL-C ≥ 1.8, in-hospital medicine (β -blockers, ACEI, ARB and statin) and in-hospital PCI.* Adjusted variables for CADPI ≥ 37 finally included age, sex, current smoking, early family history of CAD, hypertension, DM, ACS type, Killip class, HR, pre-hospital medicine (β-blockers, ACEI, ARB and statin), LDL-C ≥ 1.8, and in-hospital statin.

The subgroup analysis is shown in the forest graph (Figure 2). Pre-hospital statin treatment had no association with MACE or all-cause death in all subgroups. However, statin pretreatment showed a higher risk for severely obstructive CAD (CADPI ≥ 37) in certain subgroups, particularly in females, patients aged 50 to 75 years, and patients with hypertension.

## 4. Discussion

In the current study, we described the burden of dyslipidemia and clarified the association of pre-hospital statin treatment with CADPI and in-hospital MACE in patients with first ACS in China. We found that lipid management status was unsatisfactory and poor, particularly in the non-statin group. The regression analysis showed no association between pre-hospital statin treatment and in-hospital MACE or all-cause death in the entire population and in subgroups. Surprisingly, statin pretreatment was associated with a higher risk of severely obstructive CAD (CADPI ≥ 37) in the entire population and in three subgroups (females, those aged 50 to 75, and those with hypertension). To the best of our knowledge, this study is the first to report about the relationship of the application of pre-hospital statins with angiographic severity in China based on a nationwide registry study with ‘real-world’ settings and a nationally representative sample of patients from tertiary hospitals.

The 2007–2008 China National Diabetes and Metabolic Disorders Study showed that the prevalence of dyslipidemia was 67.43% in males and 63.98% in females in Chinese people aged 20 and older (22). Another study showed that the prevalence of dyslipidemia among Chinese adults is high, but the awareness, treatment and control of dyslipidemia are low based on a cross-sectional study on representative samples of the general population aged > 18 years from different urban and rural regions in China (23). Moreover, the updated blood lipids and statin application in the ACS population in a nationwide study in China has not been described. In the present study, 12.7% of patients had a history of pre-hospital statin treatment. The LDL-C levels were significantly lower in the statin group than in the non-statin group (2.6 ± 1.0 vs. 2.9 ± 1.0 mmol/L, *P* < 0.001). Moreover, 89.9% and 80.6% of patients in the non-statin and statin groups did not meet the lipid-lowering treatment goals (LDL-C < 1.8 mmol/L) for patients with ASCVD, respectively, and similar results were observed in the subgroups, suggesting a huge burden of potential MACEs in the future. This is a significant challenge for Chinese cardiologists, and more attention should be paid to the lipid management of patients with ACS during long-term follow-up.

Statins are highly effective for the management of hyperlipidemia and CAD (24, 25). Aside from lipid-lowering effects, statins can also inhibit platelet adhesion and thrombosis, mitigate vascular inflammation and enhance plaque stability. Statin pretreatment before myocardial ischemia attenuates infarct size and preserves myocardial function after chronic reperfusion in mice and exerts a protective effect on myocardial cells during ischemic reperfusion injury in patients with STEMI (26, 27). However, the benefits of pre-hospital statin treatment in ACS are not well established. In the current study, we found that the statin group had lower rates of death than the non-statin group. However, the regression analysis showed that statin pretreatment did not benefit patients with first ACS regarding in-hospital MACEs or all-cause death. These findings are supported by previous studies (8, 12, 28), but different conclusions were also obtained. The ARMYDA-ACS trial indicated that even short-term (80 mg 12 h before PCI) pretreatment with atorvastatin may improve outcomes in patients with ACS undergoing early invasive strategy (29). Another two meta-analyses showed similar results (30, 31). Several factors might be responsible for this difference. First, in the current study, we did not precisely address the type of statin, dose and duration, which may be attributed to the unhelpful effect of statin pretreatment for patients with ACS. Second, we only explored the in-hospital outcomes. The short time interval from onset to outcome may partly explain the negative results. Last, the study populations in the present study were patients with first ACS, which differed from previous studies. Prospective studies with long-term follow-up are necessary to further validate the results.

A previous cross-sectional study showed that statin pretreatment is associated with reduced clinical severity in patients with acute ischemic cerebrovascular events (32). However, few studies have focused on the relationship between statin pretreatment and the severity of CAD. In the present study, we found that statin pretreatment was associated with an 8.9% higher risk of severely obstructive CAD (CADPI ≥ 37). Consistent with our findings, Ndrepepa et al. (33) reported that the atherosclerotic burden was higher in patients under statin therapy. First, patients with higher disease severity prefer to use statins compared to patients with milder conditions; second, this finding may reflect the protective effects of statin therapy: statin pretreatment stabilizes plaques instead of causing rupture, thus avoiding worse outcomes, as previously described (34, 35). Notably, subgroup analyses performed based on important baseline characteristics showed the same findings: statin pretreatment was associated with a higher risk of severely obstructive CAD (CADPI ≥ 37) in females, those aged 50 to 75 years, and those with hypertension. According to a previous study, statin therapy in patients with hypertension offers a significant benefit (HOPE-3 study) (36). Moreover, statins reduce major vascular events regardless of age, but less direct evidence is available about their benefits for patients over 75 years of age (37). In addition, high-intensity statin therapy more effectively reversed coronary atherosclerosis in women with CVD than in men (38). All these findings provide support that statins might play more significant protective roles in stabilizing plaque and avoiding worse outcomes in HBP, those aged under 75 and the female population, leading to an increasing proportion of patients with severely obstructive CAD (CADPI ≥ 37).

The main mechanisms for ACS, especially fatal coronary events, is the rupture of the plaque’s protective fibrous cap. Superficial erosion, intraplaque hemorrhage, and the erosion of a calcified nodule also account for a minority (39). Thus, not only the severity of coronary artery is important, but also the stability and risk level of plaque.

It has been demonstrated that plaque statin therapies attenuate progression and strengthen plaque stability and even achieve plaque regression and it may stabilize plaque by converting its components: The ESTABLISH study clarified for the first time that statin (atorvastatin 20 mg daily) significantly reduced plaque volume (40). The PROSPECT and VIVA studies showed that the coronary plaque burden was closely related to MACE risk (41, 42). The TRUTH study also demonstrated that statin therapies reduced the fibro-fatty volume and increased the calcium volume at the same time in patients with angina pectoris (43). Another study got a similar conclusion with the TRUTH study (44). However, the PARADIGM study found that statins did not affect the progression of per centage of stenosis severity of coronary artery lesions but induced phenotypic plaque transformation (45). Taguchi et al. reported that early statin therapy reduced the necrotic core component in patients with ACS, even in the case of plaque progression (46). In the present study, we found that the prehospital statin group had a more severe stenosis score (assessed by CADPI score). There are several explanations for the seemingly contradictory conclusion: First of all, the protective effect of statin makes the plaque more stable in people with severe coronary artery disease and less prone to recurrent cardiovascular events, thus, allowing more people with severe coronary artery disease to survive. In addition, CADPI score is not as convincing as Gensini score or SYNTAX score or other intracavity evaluation methods on assessing the degree of coronary stenosis. Last, it might be attributed to the fact that people with cardiovascular risk factors, such as hyperlipidemia or carotid plaque, were more likely to have prehospital statin intervention. We did our best to control for these correlated confounding factors during the regression. Even so, this may still have influenced the conclusions. Further observations and studies on plaque phenotype and plaque burden will make the findings more convincing.

This study involved the largest nationwide registry with ‘real-world’ settings (the Improving CCC-ACS project) to investigate the relationship of pre-hospital statin treatment with the severity of disease (CADPI) and in-hospital outcomes in patients with first ACS. Some limitations of the study cannot be neglected. First, larger prospective randomized clinical trials with long-term follow-up will be necessary in the future. Second, we did not precisely address the duration, dose and class of the statins, which might have affected the results. Last, a few individuals whose basic clinical data were seriously deficient were included in this study. Hence, we did not consider people with missing data and instead used a multiple interpolation method to interpolate the data.

## 5. Conclusion

The initial ACS population in China has a huge burden of dyslipidemia with a low intervention rate. Pre-hospital statin intervention was not related to MACEs or all-cause death during hospital stay but was associated with a higher risk of increased angiographic severity in patients with first ACS. This is a significant challenge for Chinese cardiologists, and more attention should be paid to the lipid management of patients with ACS during long-term follow-up.

## Data availability statement

The data analyzed in this study is subject to the following licenses/restrictions: The original data belongs to the improving CCC (Care for Cardiovascular Disease in China) ACS project. Requests to access these datasets should be directed to the improving Care for Cardiovascular Disease in China (CCC) project.

## Ethics statement

Institutional Review Board approval was granted for the aggregate data set for research and quality improvement by the Ethics Committee of Beijing An Zhen Hospital, Capital Medical University. Participating sites were granted a waiver of patient consent under the common rule. This study was listed at www.clinicaltrials.gov (NCT02306616).

## Author contributions

LZ and ZY conceived and designed the study. JZ contributed to the design. LZ performed the study, analyzed the data, and drafted the manuscript. YZ, CC, and FD helped with the study. All authors read, critically revised, and approved the final manuscript.
