# Supplementary material for: Associations of pre-hospital statin treatment with in-hospital outcomes and severity of coronary artery disease in patients with first acute coronary syndrome-findings from the CCC-ACS project
Source: Front Cardiovasc Med. 2023 Jan 18;9:1030108. doi: 10.3389/fcvm.2022.1030108 (PMC9889368; doi:10.3389/fcvm.2022.1030108)
Supplement: Supplementary file 1 [file Table_1.pdf]

Supplementary Material

Supplementary Tables

Table S1 CAD Prognostic Index [1-3]

Extent of CAD	Prognostic Weight (0-100)
No CAD≥50%	0
1 VD 50%-74%	19
> 1 VD 50%-74%	23
1 VD (75%)	23
1 VD (≥95%)	32
2 VD	37
2 VD (both≥95%)	42
1 VD, ≥95% proximal LAD	48
2 VD, ≥95% LAD	48
2 VD, ≥95% proximal LAD	56

3 VD	56
3 VD, ≥95% in at least one	63
3 VD, 75% proximal LAD	67
3 VD, ≥95% proximal LAD	74
Left main (75%)	82
Left main (≥95%)	100

CAD, coronary artery disease; VD, vessel disease; and LAD, left anterior descending coronary artery.

1 References

[1] Mark DB, Nelson CL, Califf RM, et al. Continuing evolution of therapy for coronary artery disease. Initial results from the era of coronary angioplasty. Circulation 1994;89:2015-25.

[2] Taqueti VR, Hachamovitch R, Murthy VL, et al. Global coronary flow reserve is associated with adverse cardiovascular events independently of luminal angiographic severity and modifies the effect of early revascularization. Circulation 2015;131:19-27.

[3] Taqueti VR, Shaw LJ, Cook NR, et al. Excess Cardiovascular Risk in Women Relative to Men Referred for Coronary Angiography Is Associated With Severely Impaired Coronary Flow Reserve, Not Obstructive Disease. Circulation 2017;135:566-77.
